# Supplementary figures and images for: Aurora kinase A is essential for meiosis in mouse oocytes
Source: PLoS Genet. 2021 Apr 26;17(4):e1009327. doi: 10.1371/journal.pgen.1009327 (PMC8102010; doi:10.1371/journal.pgen.1009327)

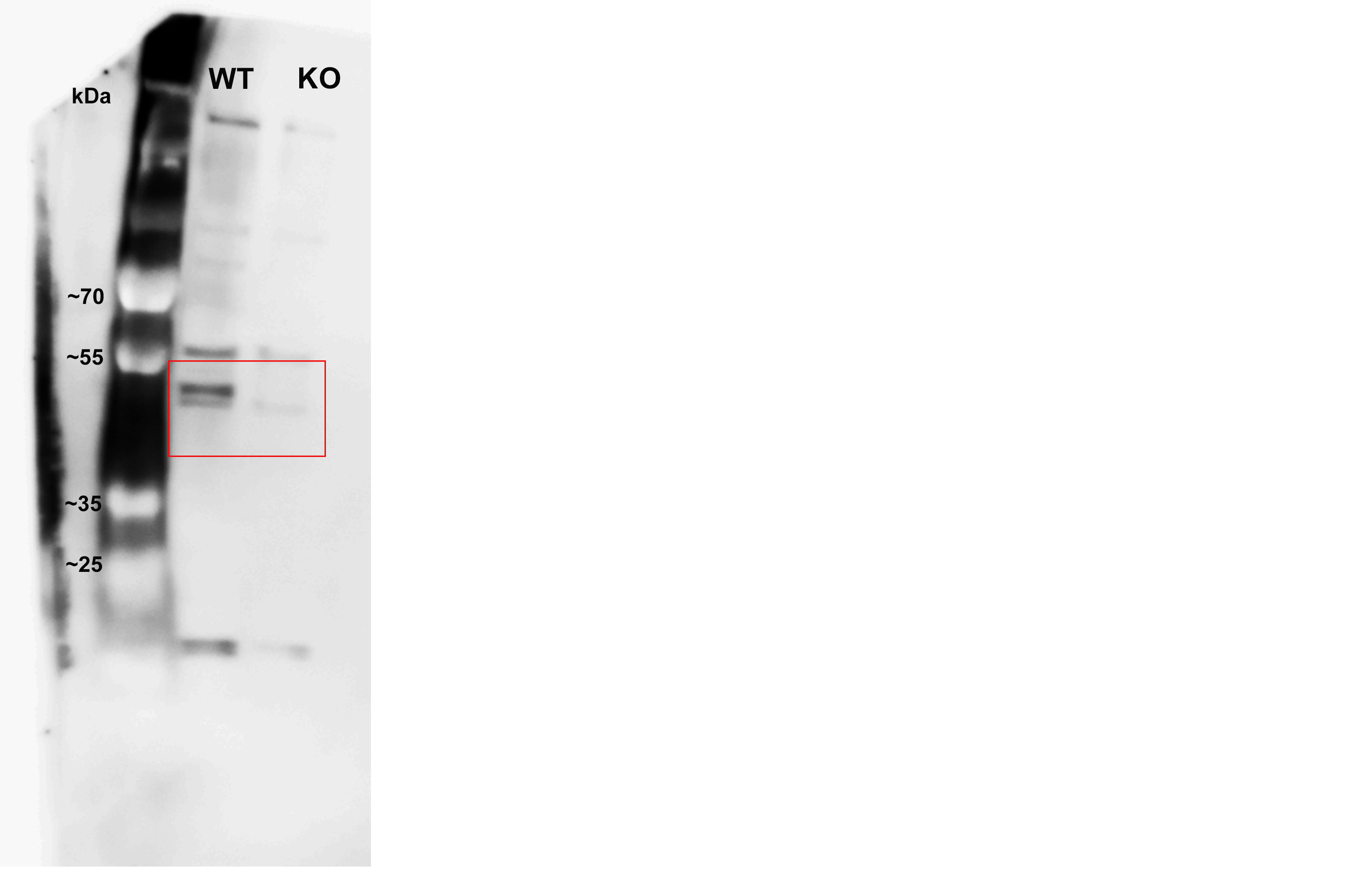

Supplement: S1 Fig — Uncropped western blot detecting AURKA from prophase-I arrested wild-type (WT) and Aurka knockout (KO) oocytes (100 oocytes/lane). Bands at ~43kDa were included in the quantifications for AURKA signal. n = 4 animals/genotype/experiment. Red box: Area showed in Fig 1A. (TIF) [file pgen.1009327.s001.tif]

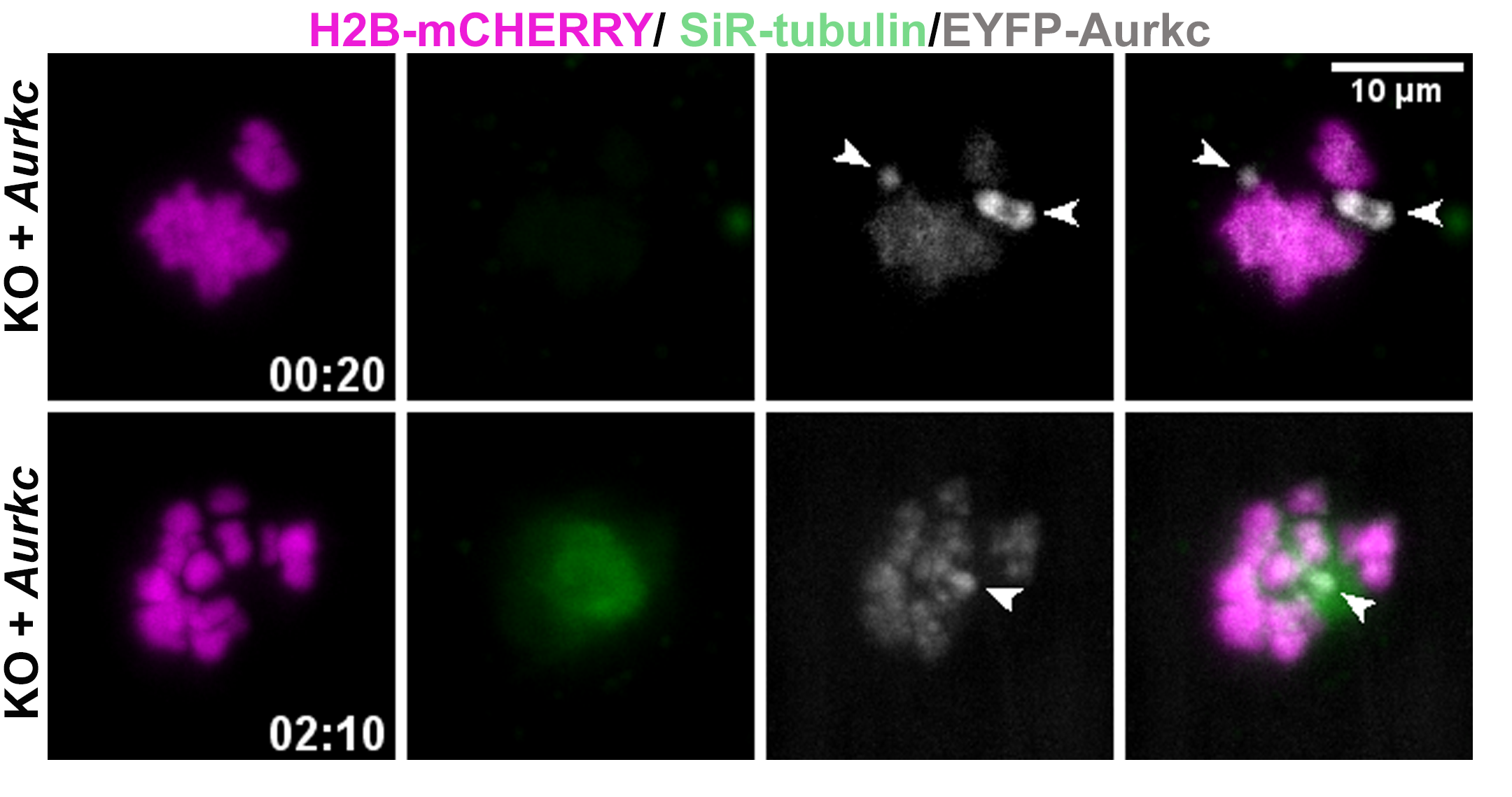

Supplement: S2 Fig — Live light-sheet imaging of KO oocytes expressing histone H2B-mCHERRY (magenta), AURKC-EYFP (gray) and stained with SiR-tubulin (green). The arrows point to AURKC localization. Maximum intensity z-projections at Metaphase I. Scale bars: 10 μm. (TIF) [file pgen.1009327.s002.tif]

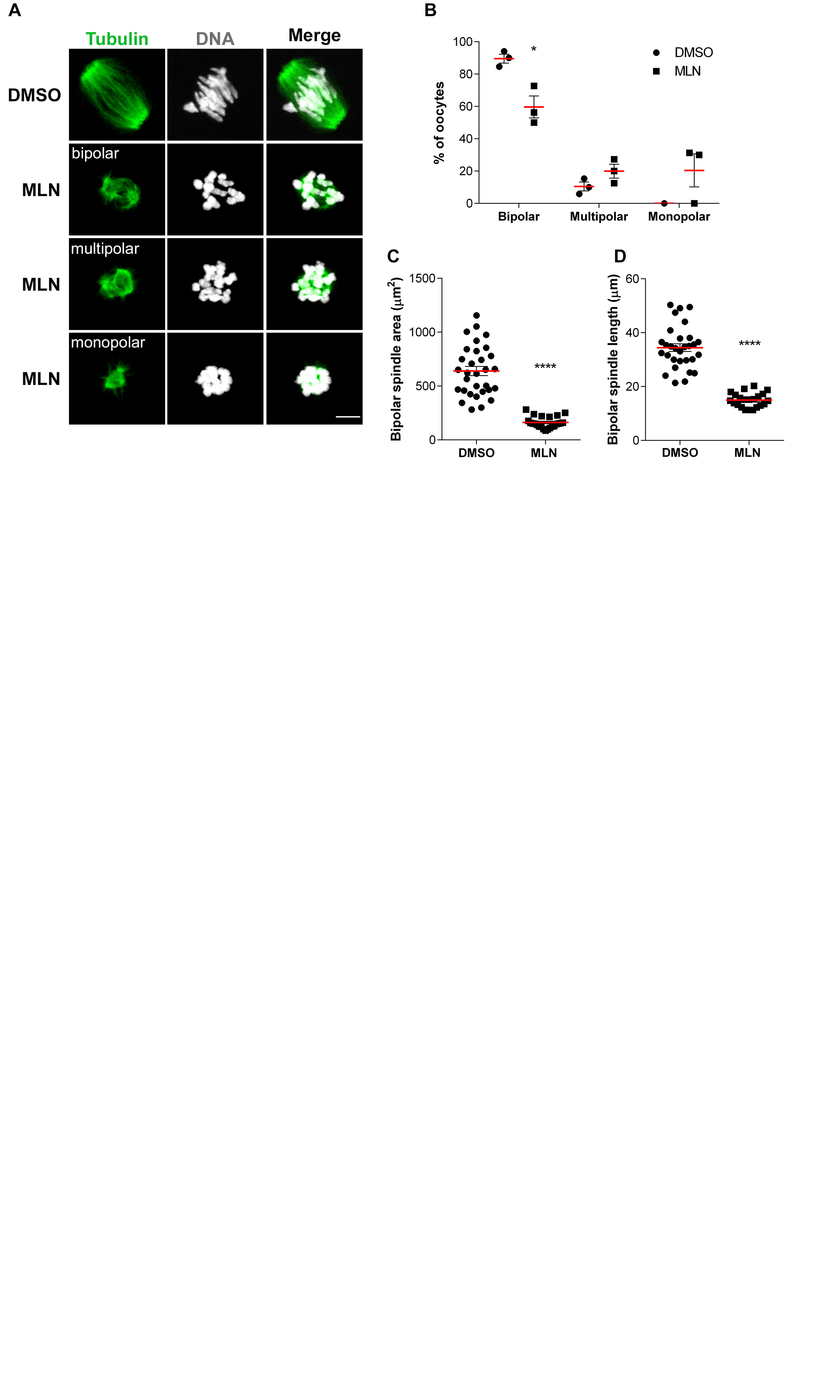

Supplement: S3 Fig — (A) Representative confocal images of oocytes at Metaphase I matured with MLN8237 (MLN) and immunostained with antibodies against α-Tubulin (green) and DAPI (gray). (B) Quantification of the percentage (%) of oocytes with different spindle phenotypes (Unpaired Students t-Test, two-tailed, * p = 0.014). (C) Quantification of the bipolar spindle area (Unpaired Students t-Test, two-tailed, **** p<0.0001; number of oocytes, WT: 31; KO: 23). (D) Quantification of the bipolar spindle length (Unpaired Students t-Test, two-tailed, **** p<0.0001; number of oocytes, WT: 30; KO: 22). Graphs show the mean ± SEM from at least 3 independent experiments. (TIF) [file pgen.1009327.s003.tif]

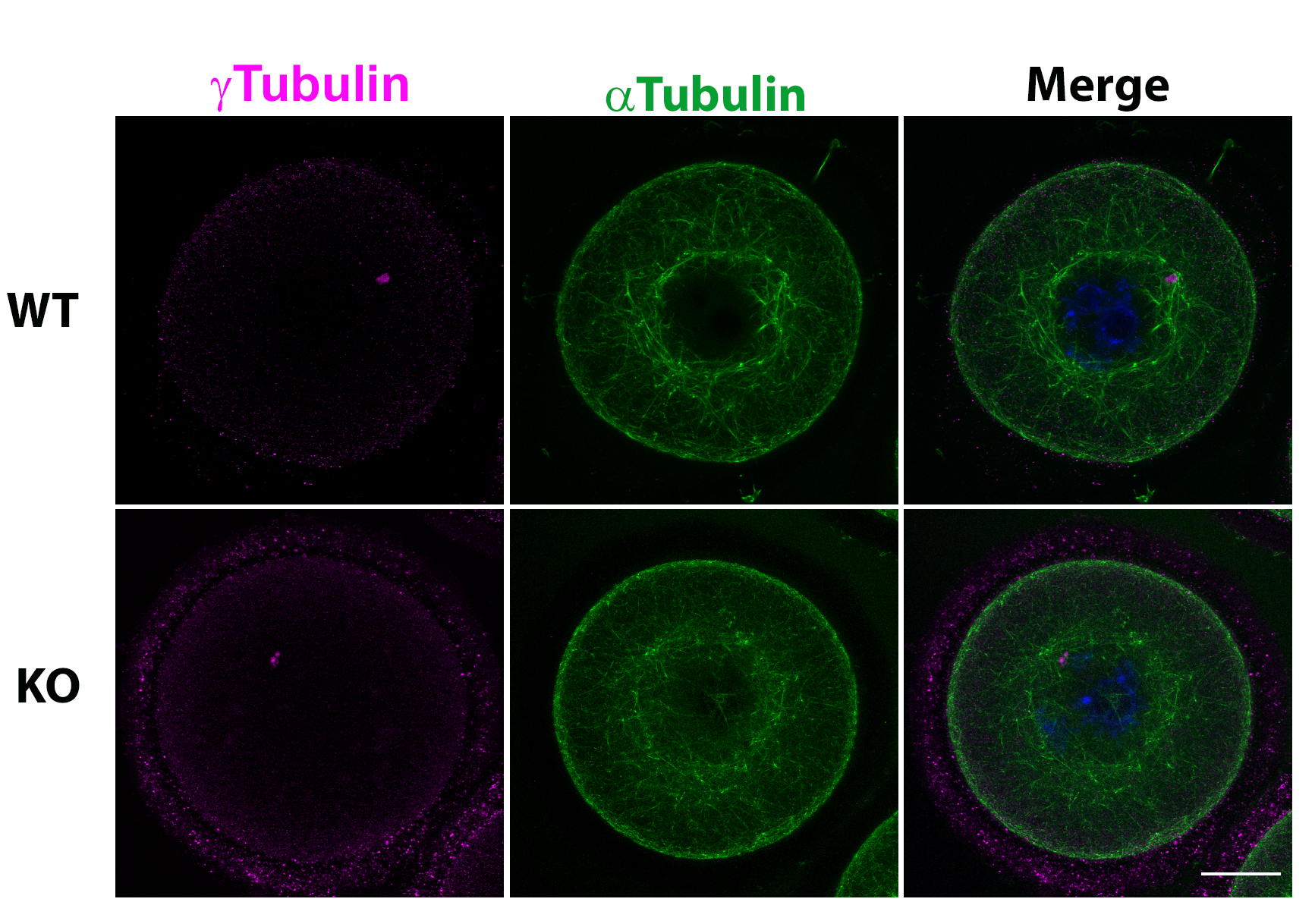

Supplement: S4 Fig — Representative confocal images of WT and Aurka KO prophase I-arrested oocytes immunostained with γ-Tubulin (magenta), α-Tubulin (green), DAPI (blue). Scale bar: 20μm. (TIF) [file pgen.1009327.s004.tif]

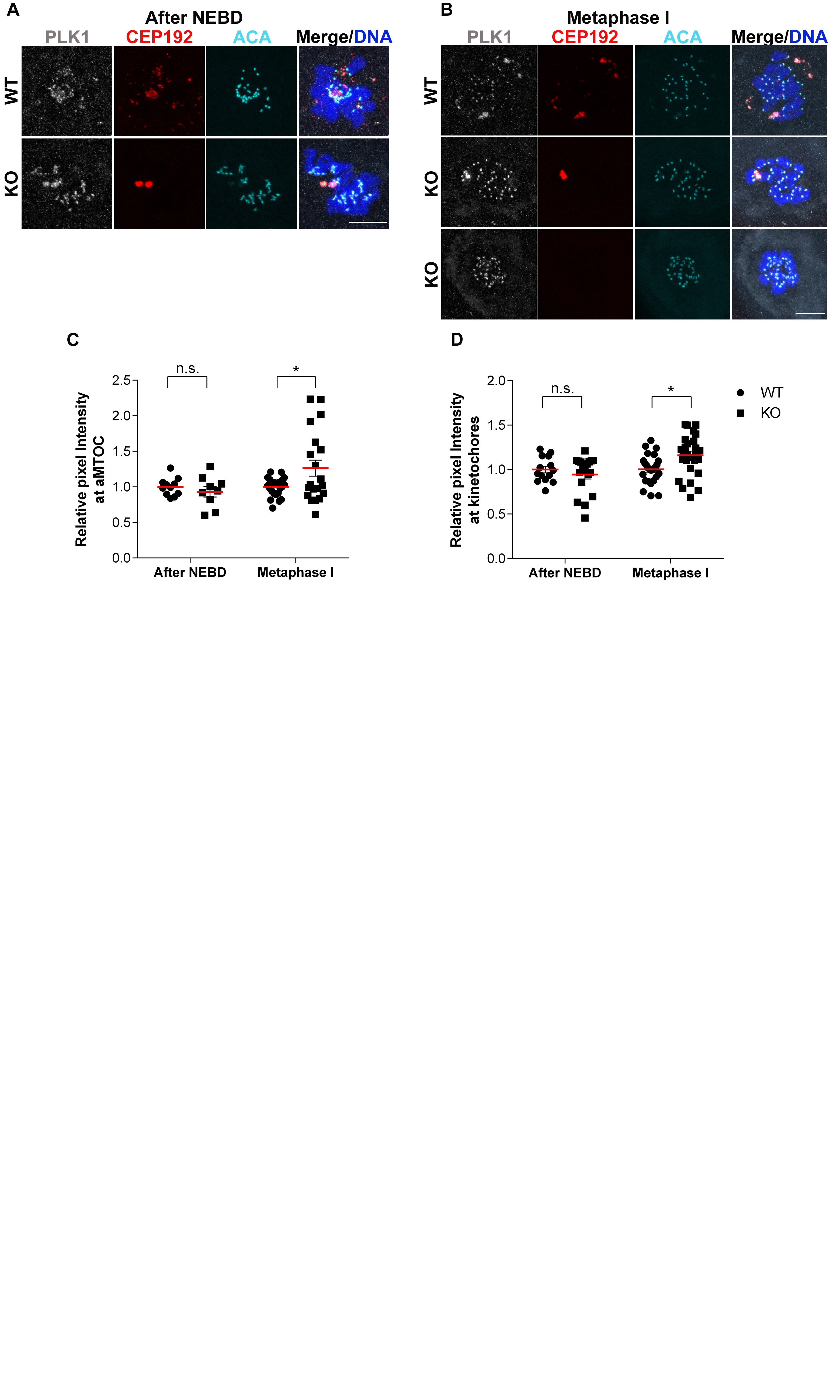

Supplement: S5 Fig — (A-B) Representative confocal images of oocytes from WT and KO females after nuclear envelope breakdown (NEBD) immunostained with antibodies against PLK1 (gray), CEP192 (red), anti-centromeric antigen (ACA; cyan) and DAPI (blue). (C) Quantification of PLK1 intensity at aMTOCs (Unpaired Students t-Test, two-tailed, p = 0.389279; number of oocytes, WT: 11; KO: 9). (D) Quantification of PLK1 intensity at kinetochores (Unpaired Students t-Test, two-tailed, p = 0.4028; number of oocytes, WT: 14; KO: 18). (TIF) [file pgen.1009327.s005.tif]

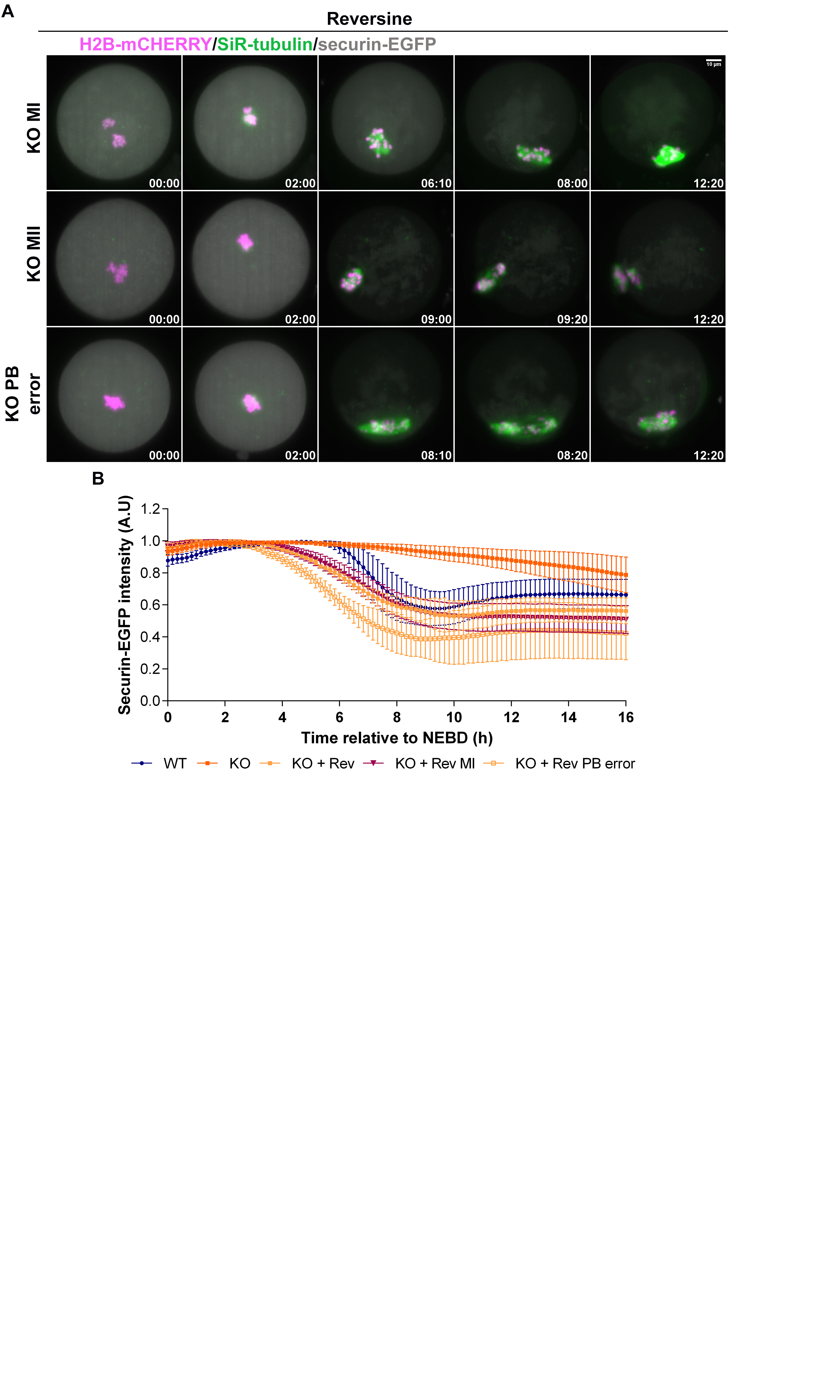

Supplement: S6 Fig — (A) Live light-sheet imaging of KO oocytes expressing securin-EGFP (grey), H2B-mCherry (magenta, chromosomes) and stained with SiR-tubulin (green, microtubules) treated with 1μM reversine. Maximum intensity z-projection images of KO oocyte arrested at MI (KO MI), KO oocyte entering Anaphase I and extruding of polar body (KO MII), and KO oocyte entering Anaphase I but had a polar body emission error (KO PB error). Time relative to NEBD. Scale bar = 10 μm. (B) Normalized intensities of cytoplasmic securin-EGFP signals. WT, KO and KO + Reversine MI groups are same as in Fig 6D. KO + Reversine and KO + Reversine PB error are split from KO + Reversine group in Fig 6D. (TIF) [file pgen.1009327.s006.tif]
