# Supplementary material for: Aurora kinase A is essential for meiosis in mouse oocytes
Source: PLoS Genet. 2021 Apr 26;17(4):e1009327. doi: 10.1371/journal.pgen.1009327 (PMC8102010; doi:10.1371/journal.pgen.1009327)
Supplement: Attachment — Submitted filename: Response to reviewers_Plos Gen_final.docx [file pgen.1009327.s011.docx]

Dear Dr. Cohen and Reviewers,

Enclosed is the revision of our manuscript entitled “Aurora kinase A is essential for meiosis in mouse oocytes” for consideration for publication in *Plos Genetics*. We are grateful for the reviewers’ expert insight and have made all modifications possible. The most significant changes you will find are removing the details about a possible role for AURKA in targeting cohesin for turnover as recommended by 2 reviewers, including aMTOC measurements from the live imaging, assessing total PLK1 and assessing C-NAP1 disengagement from aMTOCs. We find that these modifications and adjustments to the text greatly improve our manuscript. All edits are marked in tracked changes.

We regret to tell you that during the revision, our co-author Petr Solc passed away. He contributed greatly to the first version and to the revision. Our preference is therefore to keep him as a co-author. We added a dedication of the manuscript in his honor to the acknowledgements. I hope that the journal will agree to this decision and can honor our request.

Below, reviewers will find a point-by-point response to their queries and suggestions. We have copied their points and italicize them, our responses are below their points.

Sincerely,

Karen Schindler

**Reviewer 1:**

1. *The gamma tubulin images for WT are very hard to see in the downloaded .tiff images in Fig. 4A. (first panel)…*

We agree that it is difficult to view γ-Tubulin foci in WT oocytes, in part because they fragment and are small in early Pro-metaphase. We have added red arrows to highlight the foci better.

2. *The authors describe PLK1 as not being phosphorylated (pT210). However, detailed assessment of PLK1 localization and PLK1 phosphorylation is required for this study.* *Do the authors see defective PLK1 phosphorylation at the kinetochores as well as at the MTOC? What is the general status of PLK1 localization in the Aurka cKO.*

These points were great suggestions. We conducted 2 new analyses that are added to the manuscript. First, we assessed pPLK1 at kinetochores. We conducted this analysis at pro-Metaphase I and at Metaphase I. We show in new Figure 5E, F and H, that pPLK1 at kinetochores is reduced. The reduction is not as much as the reduction measured at aMTOCs (~40% vs ~70% reductions). We reason that there is some reduction at kinetochores because there is likely kinetochore-localized AURKA in oocytes as in HeLa cells (DeLuca KF, et al. J Cell Biol. 2018;217(1):163-77.). The description of this data can be found in lines 301-306 where we state:

“But, surprisingly, pPLK1 levels were also significantly reduced by ~40% at kinetochores in *Aurka* KO oocytes (Fig. 5C, E-F, H) despite an apparent increase in total localized PLK1 by Met I (Fig. S5B, D). The reduction of chromosomal pPLK1 likely reflects an undetectable population of AURKA at kinetochores, a population that has been documented in mitotic cells [40].”

We next assessed total PLK1 levels to ensure that the reduction in pPLK1 wasn’t due to a reduction in total PLK1. In a new supplemental Figure S5 we show PLK1 at prometaphase I (Fig. S5A, C) and Metaphase I (Fig. S5B, D). We show that there is no difference in total PLK1 at aMTOCs or at kinetochores in early prometaphase. Interestingly PLK1 levels increase at aMTOCs and kinetochores at Metaphase I. We do not know why total levels would increase, but, importantly, the data support the model that phosphorylated PLK1 decreases in an AURKA-dependent manner. This information can be found in lines 298-301:

“Consistent with our hypothesis, PLK1-T210 phosphorylation was significantly decreased in *Aurka* KO oocytes at aMTOCs by ~60% (Fig. 5C-D, F-G) at both stages; however, levels of total PLK1 at aMTOCs remained unchanged at NEBD and increased at Met I (Fig. S5A-C).”

3. *AURKC is included in the model in Fig. 7. Can AURKC phosphorylate PLK1? It would be very informative to compare a Plk1, Aurka double KO Vs the Aurka (and Plk1) CKO (or inhibition of PLK1) to determine the overlap and unique roles of these two cell cycle kinases.*

We agree that AURKC targeting of PLK1 is interesting, but there are several technical reasons why we cannot complete this analysis at this time. In AURKC KO oocytes, AURKA compensates for its loss precluding the ability to assess AURKC’s contribution in this way. If we try to inhibit AURKC, we also inhibit AURKB. Furthermore, we do not have a PLK1 KO strain to create double KO lines. As we continue to develop new methods to get at these functions, we hope to revisit this idea in future studies. We have elected to describe this possibility in the text found starting in line 458:

“It is not known if AURKC can phosphorylate PKL1, but given that some phosphorylation persisted, it is likely that PLK1 is a shared substrate, a possibility that is worth future investigation.”

4. *In PLK1 inhibition studies by one of the co-authors gamma tubulin is absent from MTOCs. This is different from what is reported in this study. If this is the case, then some discussion is needed. In general, the contrast between Plk1 and Aurka phenotypes is needed. The authors suggest they are similar, but there are clear differences*

We have added lines to the discussion to clarify what is different in these scenarios. These details are found starting at line 465:

“However, when PLK1 was inhibited in mouse oocytes, γ-tubulin and pericentrin, were absent from aMTOCs, suggesting that PLK1 is needed to maintain the composition of these structures [62]. Phenotypic differences exist between inhibition of PLK1 and our knockout oocytes. Here, we show that only 27% *Aurka* KOs lack an aMTOC close to the spindle, whereas inhibition of PLK1 abolished MTOCs. This difference can be explained by complete inhibition vs the 60% reduced activation at MTOCs in the *Aurka* KO background. In KO oocytes with aMTOCs, they were smaller and hyper-condensed compared to WT aMTOCs. Although PLK1 and AURKA are part of the same pathway controlling aMTOC fragmentation, there are some PLK1-specific functions; such as, chromosome condensation by regulating localization of condensin and cohesin [38]. *Aurka* KO oocytes can condense the chromosomes, and although they are delayed in resolving individual chromosomes during meiotic maturation, they eventually do.”

5. *The authors need to assess localization of the inter-MTOC linker, C-NAP1, to determine whether Aurora A is required for timely disassociation from MTOCs.*

We appreciate this suggestion to strengthen the role in fragmentation. We conducted C-NAP1 detection and analysis and find that it persists after NEBD in oocytes lacking AURKA. This data is in revised Figure 5I-K. Details of the data are found in lines 307-314:

“An essential step for aMTOC fragmentation is the dissociation of the centrosomal cross linker protein C-NAP1 from aMTOCs, a step which requires PLK1 activity [12]. We next evaluated C-NAP1 localization to confirm PLK1-dependent fragmentation defects. At prophase I, both WT and *Aurka* KO oocytes contained aMTOC-localized C-NAP1 (Fig. 5I, K). Consistent with reduced PLK1 activity, C-NAP1 persisted at aMTOCs after NEBD in *Aurka* KO oocytes whereas it disappeared in WT oocytes (Fig. 5J-K). These data suggest that AURKA regulates MTOC fragmentation by phosphorylating and thereby activating PLK1 after NEBD.”

6. *The authors could analyze the nature of the spindles that are present as either monopolar or bipolar in more detail. Can they recover from nocodazole (or similar) treatment? Are the spindles able to reform? Where are the spindles developing from?*

In the original submission, we quantified monopolar and bipolar spindles from the fixed images (please see Figures 4C, I) and assessed the time it took for spindles to become bipolar in live imaging (please see Figure 4H). To determine if spindles oscillate between mono and bipolar states, we re-visited our live images and looked at later time points. We found that 73% of the Aurka KO oocytes had stable spindle phenotypes and did not oscillate between mono/bipolar after they reached Met I. This description is found in Lines 283-286:

“Moreover, after WT oocytes progressed past Met I, we observed that ~73% of the *Aurka* KO oocytes maintained their spindle configuration. Therefore, the monopolar spindle defects observed at Met I were not due to a delay in spindle building but rather was a terminal phenotype.”

At this point, we do not know if the spindles re-form after nocodazole treatment or where they develop from. We envision analysis of spindle formation in AURKA-independent pathways to be a detailed, follow-up study.

7. *In Fig. 5K they show an example where PCNT is completely absent in an Aurka CKO oocyte. Is this common? Has this been quantified?*

We quantified this point and find that 27% of AURKA KO oocytes do not have aMTOCs. This quantification is included in the text on Line 318:

“In contrast, some *Aurka* KO oocytes lacked a visible aMTOC (27%) or others had one hyper-condensed spindle pole, with reduced volume and width (Fig. 6A-C).”

8. *The authors should discuss the reported link between Plk4 and Aurora A …*

We have included this link in the revised discussion. The text starting at Line 488 reads:

“Another explanation could be a partial compensation by PLK4 that has been reported to be present at aMTOCs in mouse oocytes and works with AURKA to initiate microtubule nucleation [27].”

9. *The authors state that “To our surprise, although reversine-treatment restored securin-EGFP destruction in Aurka KO oocytes (Fig. 6D-E), 64% of these oocytes did not enter Anaphase I and did not extrude a polar body”. Is this really a surprise given the spindle defects described earlier?*

We do think this is a surprise. The SAC detects kinetochores not attached to microtubules (not spindle defects). While we did not directly assess attachment status, there was no indication in live imaging or in fixed Metaphase images that any bivalent was not attached to microtubules. At least half of the KO spindles were bipolar (50%), albeit small. Therefore, by removing the signal that may be sensing reduced tension in a small spindle, we anticipated at least 50% to undergo Anaphase onset.

10. *The authors state that AURKA regulates cohesin removal… in an APC/C independent manner. … Nevertheless, as it stands, this observation is somewhat distracting from the main story of the paper.*

The consensus of Reviewers 1 and 3 is that the cohesin data is distracting and preliminary. We have elected to remove this part of the manuscript to keep our message clear and concise. We hope to follow up on this interesting finding in future studies.

11. *The authors description of Gdf9-Cre expression and deletion of critical exons of Aurka should be better explained (lines 140 to 144).*

The information about the KO has been updated in the methods (Line 513). During the revision, a manuscript on male meiosis using the same AURKA KO mouse line was published in EMBO Reports (Wellard et al). In this manuscript, they provide additional details of the deletion allele and we include this citation at line 142. Details about Gdf9 expression timing follows this citation.

12. *Line 260 – “the dominate” needs to be corrected.*

This typo has been corrected.

13. *Error bars are missing from Fig. 3F.*

We have now included 95% exact confidence interval bars in the figure.

14. *The authors should comment on the general morphology of the bivalents. They essentially appear to be WT for chiasma, size, etc. but this is not reported in the paper. Was there an indication of loss of association between homologs in the mutant?*

We did not observe any abnormalities in morphology of the bivalents. We added this detail to line 232:

“The chromosome morphology appeared normal, and we did not observe a loss of cohesion between homologs or sister chromatids.”

15. *At times it is hard to distinguish between channels. For example, Fig. 5A is hard to distinguish between the CDK5RAP2 signal. Same for the zoom in Fig. 5B. It would be great to have separated channels more often.*

We have modified this figure to separate the channels as suggested.

**Reviewer 2**

1. *In Figure 2, the graphs show a decrease in number of corpus luteum at both 2 months and 6 months in the KO compared to the WT. However, only the data at 2 months was statistically significant. The text states that there is a difference in CL and does not specify at which age. The authors should state that differences occur at 2 months and 6 months, but only the data from 2 months was significantly different from WT.*

We have added greater clarity to the text starting at line 175:

“… *Aurka* KO ovaries from females that were two months old had about one-half of the number of CLs in comparison to WT. Furthermore, although not statistically significant, *Aurka* KO ovaries from older females (six months) also had reduced number of CL (Fig. 2C-F) suggesting that not all oocytes in fully developed follicles were ovulated.”

2. *Figure S1 does not show the SiR-tubulin, as the figure legend states. It is hard to demonstrate that the AURKC is at the spindle poles without the Sir-tubulin.*

We apologize for the omission. The revised figure (now S2) includes the Sir-tubulin channel.

3. *When describing Fig. 4B, the authors mention that the chromosomes in the KO oocytes do not resolve from one another in early pro-Met I. However, they are basing pro-Met 1 on the timing of the wildtype oocytes. From the images and graph, it may be more accurate to say that the KO oocytes are delayed in the time of chromosome*

We thank the reviewer for this suggestion. We included this point in Line 229:

“In contrast, the chromosomes in the majority of *Aurka* KO oocytes did not resolve from one another at early pro-Met I but they did later, suggesting that chromosome resolution in *Aurka* KO oocytes is delayed (Fig. 4B).”

4. *In the KO, do more oocytes form bipolar spindles at later time points beyond the 7hr timepoint? Are the cells with monopolar spindles delayed in bipolar spindle assembly or are they unable to form bipolar spindles?*

We re-evaluated our videos to assess stability of bipolar spindles or progression of a monopolar into a bipolar spindle with time. We found that 73% of the *Aurka* KO oocytes had stable spindle phenotypes and did not oscillate between mono/bipolar after they reached Met I. This description is found in Lines 283-286:

“Moreover, after WT oocytes progressed past Met I, we observed that ~73% of the *Aurka* KO oocytes maintained their spindle configuration. Therefore, the monopolar spindle defects observed at Met I were not due to a delay in spindle building but rather a terminal phenotype.”

5. *When describing securin degradation, the authors fail to mention that it is substantially delayed in the KO oocytes with reversine. Although the authors are correct in highlighting the fact that securin is degraded, showing that the APC/C is activated, the delay in activation could show an important aspect to APC/C regulation by AURKA.*

Thank you for pointing out this detail. We added a detail to Line 379 to indicate the 2h delay:

“…restored securin-EGFP destruction in *Aurka* KO oocytes, this restoration was almost 2h slower than in WT (Fig. 7D-E)”

6. *Similarly, the authors should also mention the delayed time to anaphase onset in the KO with reversine in 6G (they only mention the WT).*

We added this detail of a 4h delay in anaphase onset to the text at Line 379:

“Thirty-six percent of *Aurka* KO oocytes treated with reversine entered Anaphase I, but there was a ~ 4h delay compared to WT oocytes treated with reversine, (Fig. S6A, B; Movie S4)…”

7. *The reduction of Rec8 by 35% suggests that separase is active for Rec8 cleavage. And, if there is some separase activity, the chromosomes should eventually segregate, perhaps after a long delay. However, is the Rec8 protected from full cleavage in the KO? For example, is SGO2 protection spread to the whole chromosome (and not just the pericentromere) in the KO oocytes, preventing Rec8 cleavage?*

Reviewers 1 and 3 found the Rec8 portion of the manuscript preliminary and distracting. We have therefore elected to remove this part of the manuscript. But, this is an interesting idea to explore for future work in this area.

8. *Line 423 may be too strong, “….we show that PLK1 is not activated in Aurka KO oocytes”. The authors show reduced phosphorylation, not absent.*

We have modified this sentence:

“… we show that PLK1 activity is reduced in *Aurka* KO oocytes.”

9. *Lines 118 and 167 words missing*

We have edited these omissions.

**Reviewer 3**

1. *Line starting 171 – if Aurka KO ovaries had 50% reduction in the number of CL in comparison to WT (Fig. 2C-F), how do the authors know the reduced fertility of AURKA KO mice is due to diminished ovulation and not because of any of the meiotic phenotypes they observe?*

Our purpose in performing the histology was to make sure that the mice in fertility trials ovulated by assessing presences of corpus luteum (CL). When we saw reduced numbers of CL, we worked back in time to make sure there weren’t issues with the ovarian reserve. Granted, any oocyte ovulated at Met I will should not give rise to a healthy embryo or live pup and therefore ovulating Met I oocytes alone, no matter how many, will account for infertility. We have tempered the rationale as suggested and explained our reasoning of conducting histology as described here. Please see edits in Lines 167 and 169.

2. *Fig 3A-B – could the polar body extrusion phenotype be associated with hormonally-forced ovulation of oocytes in KO mice that would not have been ovulated? There is 50% reduction of ovulation in these animals after all.*

Because 100% of the oocytes arrest at Met I in both hormonally primed and unprimed mice (we do not prime mice 3-4 weeks of age, which were sometimes used here), we are confident that the hormone is not causing the PBE phenotype. As the reviewer points out, there is a reduction in CL (ovulation), so by priming the mice, we are likely pushing more oocytes to be ovulated than would naturally occur. Because we don’t see gross defects in oocyte quality, we do not have any indication that this approach is causing the phenotype. We include a comment in the results to direct readers to a recent publication indicating no effects in mouse oocytes from hormone treatment (Line 183-186):

“To harvest cells from oviducts, we had to induce ovulation through hormonal stimulation, which was not used in the fertility trials or histology. To our knowledge, hormone stimulation does not negatively impact meiotic maturation (https://pubmed.ncbi.nlm.nih.gov/31630300/).”

3. *Lightsheet imaging of oocyte meiosis is a big effort that should provide very high temporal resolution. It is thus disappointing that the authors do not provide better movie stills in Figure 3 of the entire meiosis I process but instead opt for showing only distinct stages of meiosis I.*

We agree with the effort for getting this imaging going and are grateful that Petr Solc initiated this type of imaging in our field. As we indicated in the cover letter, Petr passed during the revision and his lab work is frozen until administration items can be solved. We have done our best with the movies we had to improve the quality of the stills by adding additional time points (Figure 3E) and separating channels (Figure 5A). We hope these modifications satisfy the reviewer and that take our challenging situation into consideration.

4. *The multiple stages of meiosis I in Figure 1E should be thoroughly quantified and compared between the different conditions and the method of analysis should be provided.*

Unfortunately, we are not certain what the reviewer is referring to. Figure 1E is the pCDC25 data and does not contain different stages of meiosis. If they are referring to Figure 3E, we have added additional stills which highlight meiotic stages. Only WT and KO + AURKA oocytes mature past Metaphase I. Therefore, there is nothing additional to quantify to add to the story.

5. *What are the technical limitations mentioned in the manuscript that prevent imaging of oocyte after induction of meiosis I?*

The technical limitation reflects the time between washing out the meiotic resumption inhibitor and setting up the imager. The Solc team does not have a microfluidics chamber to allow for media exchange. Therefore, the movies begin shortly before NEBD. Based on the suggestion, we revisited the movies and found that we could extract more information than we originally thought (see below).

6. *Overall, from the supplementary movies it appears that the major spindle assembly defects could be MTOC lack of stretching/fragmentation followed by bipolarisation defects. Can the authors perform better quantification of stretching/fragmentation by taking advantage of the lightsheet imaging they have done (e.g. volume measurement over time of MTOCs)?*

Using Imaris imaging analysis, we measured the area of the major aMTOC focus in each oocyte from WT and KO animals overtime (new Figure 5B) and track the number of aMTOCs in these oocytes. In WT oocytes the number of aMTOCs first increases, reflecting fragmentation, and then decreases, reflecting coalescence. In KO oocytes, this number stays flat reflecting a loss of fragmentation. In WT oocytes the area of the major aMTOC only increases during coalescence and stays flat in KOs. This information is described starting at Line 274-282:

“As a result of fragmentation, we observed a reduction in the median size of the major aMTOC and a subsequent increase in aMTOC numbers (Fig. 5A-B). As WT oocytes transited from pro-Met I to Met I, aMTOCs sorted, spindles elongated and finally MTOCs coalesced to form two spindle poles. During this time, there is a measurable increase in aMTOC size and a reduction in aMTOC foci numbers (Fig. 5A-B). In contrast, and consistent with our previous result (Fig. 4A), *Aurka* KO oocytes lacked multiple cytoplasmic aMTOCs and had only 1-2 large aMTOCs at the time of NEBD (Fig. 5B). *Aurka* KO oocytes never fragmented the large aMTOC (0/16 KO vs 12/12 WT) and therefore both the size and numbers remained constant (Fig. 5A-B; Movie S2).”

7. *These two processes are motorized – stretching/fragmentation by dynein and bipolarisation by kinesin-5. How are these motors affected in AURKA KO oocytes? while the PLK data demonstrate that aMTOCs are largely inactive (low microtubule mass in prometaphase), this should not fully affect at least bipolarisation since chromatin-mediated microtubule nucleation can eventually assemble microtubules. This suggests other spindle assembly factors such as kinesin-5 may also be affected in AURKA KO oocytes.*

We appreciate the reviewer’s thoughts and curiosity. Determining all the mechanisms at play is beyond the scope of this one manuscript. However, we did decide to take a look at kinesin-5 (EG5; KIF11). This kinesin is a known substrate of AURKA, although, they function of AURKA-phosphorylated KIF11 is not described in the literature, to our knowledge. We found that total KIF11 signals were increased in KOs and localized to aMTOCs. This increase in signal likely reflects the size of the major aMTOC that failed to fragment. We decided to present this data in revised Figure 6H-I. If it is not phosphorylated in the KO (we assume it isn’t, but no antibody exists to demonstrate this), then phosphorylation does not affect its localization but likely its function. We include this speculation in Lines 331-339:

“In mouse oocytes, kinesin-5 (Eg5; KIF11) is required for spindle bipolarization through sliding anti-parallel microtubules apart to facilitate separation of aMTOC fragments [12], and its inhibition causes monopolar spindles. We therefore examined KIF11 at Met I aMTOCs. We observed nearly a two-fold enrichment of KIF11 levels at spindles poles in *Aurka* KO oocytes compared to WT (Fig. 6H-I), an increase which likely reflects the spindle pole size. Therefore, loss of AURKA does not affect KIF11 localization. Because KIF11 is an AURKA substrate it is possible that this enrichment reflects an accumulation of KIF11 in a non, or reduced function state.”

8. *How does loss of AURKA affect cohesion?... It might be beneficial to significantly shorten this section or even leave it out as at this point it does not provide much insight about AURKA function… Unless the authors wish to go into mechanistic details that address these issues, the Rec8 section should be revised. I would say this is a topic for an independent study and takes attention away from the unique function of AURKA at aMTOCs.*

We agree with the points raised here and because of the general agreement amongst reviewers, we removed this section. In our model (Fig. 8) we leave a role for AURKA at anaphase marked with a “?” since reversine failed to fully rescue the arrest phenotype.

9. *I would argue that the mouse oocyte spindle pole being a liquid-like or phase-separated structure is still a model that requires further research before it is accepted in the field…. rephrasing this should not affect the main message of the paper.*

We apologize for the oversight on using the term LISD as we did not consider the “newness” of this topic in our writing. We removed this term throughout and simply refer to TACC3 localization on the spindle being altered.

10. *Line 78 – centrioles ‘are’ lost. Even better to use the term ‘eliminated’ instead since at least in other species this is an active process. It would also be good to cite here how centriole elimination in oocytes is conserved across several species from flies, echinoderms to mammals.*

We have edited this line (75-77) as recommended. It now reads: “However, in mammalian oocytes this process is strikingly different because centrioles are eliminated during oocyte development [4-6]. The elimination of centrioles in oocytes is conserved across taxa from flies, echinoderms to mammals, and it is critical to ensure the sole inheritance of sperm centriole in the zygote [5, 6].

11. *…introducing MTOCs as aMTOCs instead…*

We have modified the references throughout.

12. *Line 82 – better to stick here with more widely used terms such as MTOC coalescence here*

We modified our wording here.

*13.* Line 89 – the SAC can be fulfilled by ‘inappropriate’ attachments that still cause tension so for accuracy, better to write ‘until all kinetochores are attached to microtubules’.

We clarified this point as indicated.

14. *Figure 1 – please refrain from quantifying depletion from ECL blots. The blots in figure 1A are pretty clear that the deletion has worked. Could the authors also provide uncropped blots in the supplementary data as well?*

We have removed the quantification and provide a new supplementary Figure S1 with the uncropped blot.
